# Self-compassion training and psychological well-being of infertile female

**DOI:** 10.18502/ijrm.v17i10.5300

**Published:** 2019-11-07

**Authors:** Seyed Alireza Afshani, Azade Abooei, Ali Mohamad Abdoli

**Affiliations:** ^1^Social Welfare Department, Social Sciences Faculty, Yazd University, Yazd, Iran.; ^2^Islamic Azad University, Yazd Branch, Yazd, Iran.; ^3^Research and Clinical Center for Infertility, Yazd Reproductive Sciences Institute, Shahid Sadoughi University of Medical Sciences, Yazd, Iran.

**Keywords:** Psychological, Infertility, Female.

## Abstract

**Background:**

The empowerment of psychological well-being is an important and fundamental issue among infertile females.

**Objective:**

The present study investigates the effect of teaching self-compassion on the psychological well-being of initial infertile women.

**Materials and Methods:**

In this cross-sectional, quasi-experimental study with pre-test and post-test, 32 infertile women who were referred to the Yazd Reproductive Sciences Institute during 2016-2017 were enrolled. The participants were randomly divided in two groups as control and experiment (n = 16/each). The participants only in the experimental group received 8 sessions of 90 min training (Self-Compassion Training). Ryff's psychological well-being questionnaire was applied (reliability coefficient = 0.82) and covariance analysis statistical test was used to test the research hypothesis.

**Results:**

There was a significant difference between the estimated mean scores for improving the psychological well-being of the participants in the experiment and control groups (p = 0.007), and the difference indicates that 72.7% of the covariance of the post-test scores is due to self-compassion intervention. Therefore, the intervention of self-compassion training affects the improvement of psychological well-being among infertile women. Also, the pre-test variable is significant with the effect of 94.2% (p = 0.006).

**Conclusion:**

The findings showed that teaching self-compassion to initial infertile women has an effect on their psychological well-being.

## 1. Introduction

Infertility is a sensitive and difficult issue for spouses, especially for those who have been married for a long time (1). Spouses who are facing with infertility have various emotional responses, including low self-esteem, anger, sadness, jealousy of other couples with child, anxiety, and eventually, depression (2). in Iran, childbearing as an important part of females' identification has a central role in their social life. Women who are confronted with infertility have more problems in comparison with men and are also more vulnerable to infertility (3). Moreover, According to the estimates by the World Health Organization and the statistics of the Iranian Infertility Centers, between 10 and 15% of couples who have got married for 1-5 yr, are faced with infertility, which affects both the partners therefore, involved people are confronted with many problems. This problem is the third most important reason for divorce due to this fact that, they consider childbearing as a source of maintaining the foundation of the family, survival, and social well-being (4). Also, medical intervention can greatly affect the quality of the lives of spouses and their needs such as emotional, social, physical, sexual, intellectual, and psychological well-being (5). It may become the greatest obsession in their lives and reduce the level of marital satisfaction and cause the family separation (6). Recently, concepts such as psychological flexibility, self-compassion (SC), experiential avoidance and kindness, which have some effects on psychological well-being, have been addressed as important psychological processes (7). These concepts are derived from the third wave of therapies. Pieces of evidence suggest that these processes can significantly reduce the mental sufferance of difficult situations (8). SC as a term was first introduced by Neff in 2003 (9). SC began with this theory that all human beings are valued and respected, regardless of the degree of progress and physical and social interests. "It involves the following three main components: (a) self-kindness (understanding oneself toward self-criticism), (b) common humanity (considering one's experiences as part of the larger human experience rather than regarding them as separating and isolating), and (c) mindfulness (maintaining painful thoughts and feelings in balanced awareness rather than over-identifying with them). Since infertile women often feel frustrated and lonely and criticize themselves (10)", they are reluctant to accept their painful feelings (11), therefore, the researcher have chosen the term SC for the intervention.regarding to infertile females condition, they experience certain psychological problems such as anxiety, depression, shame, self-criticism, and self-judgment, and also considering that many studies show the influence of compassion training on releasing negative emotions, the researchers have decided to examine the effectiveness of SC training on the psychological well-being of infertile women in this study.

## 2. Materials and Methods

In this pre-test and post-test, quasi-experimental cross-sectional study, 32 initial infertile women referred to the Yazd Reproductive Sciences Institute during 2016-2017 were enrolled in two groups: the control, and the experimental groups (n=16/each). The experimental group received eight 90- minutes training sessions with SC concepts including mindfulness, self-kindness and common humanity, and improving mental well-being. The aim of these concepts training was to prevent the self-blaming and self-criticism prevention among infertile women. The content of these trainings is are shown as follows (table I).

**Table 1:** Contents of training session


**Sessions**	**Contents**
I	Explain about self-compassion and its necessity for infertile female + pre-test
II	Introduction of self-compassion component including self-kindness, mindfulness, and common humanity
III	Mindfulness exercises like meditation and relaxation with concentration on inhale and exhale
IV	Explanation about automatic thoughts and watching them without believing them
V	Self-acceptance and having unconditional love toward themselves
VI	Working on inflexible habits and dependency
VII	Having exercises about being mindful and living in the present moment
VIII	Summarizing all sessions information

### Inclusion and exclusion criteria

• Primary infertility
• Age < 35 years
• More than two absences in training sessions
• Failure to do homework more than three times

### Psychological well-being Ryff

A theoretical model of psychological well-being includes six particular dimensions of wellness (Autonomy, Environmental mastery, Personal growth, Positive communication with others, Purpose in life, Self-acceptance), has two forms; short & long one that in this research, the long form which includes 84 items was used (11).

### Ethical consideration

The study was approved by the ethical & research committee of Yazd Reproductive Sciences Institute, Shahid Sadoughi University of Medical Sciences, Yazd, Iran (IR.SSU.RSI.REC.1395.27). The study was carefully explained to the participants and informed consent was obtained before being recruited into the study. The participation in this research was entirely voluntary. in addition, they were given permission to leave the group in any time that they want.

### Statistical analysis

Data were analyzed using Statistical Package for the Social Sciences (SPSS software), version 22.0, SPSS Inc, Chicago, Illinois, USA. In addition to descriptive statistics, the analysis of covariance (ANCOVA) was used to analyze the data.

## 3. Results

There was no significant difference between the pre-test scores in the control and experimental groups. Furthermore, the difference in the mean scores of the control group was low in the pre-test and post-test (3.5 points), but in the experimental group, the difference between the mean scores in the pre-test and post-test was relatively high (Table II). There was not a significant difference between the mean value of psychological well-being among infertile women in the control group in the pre-test and post-test (p = 0.15), whereas, in the experiment group, this difference was significant (p = 0.01). Also, the mean value of psychological well-being of infertile women did not show a significant difference between the control and the experiment groups in the pre-test (p= 0.98), while in the post-test, this difference was significant (p= 0.04) (Table III).

### Examining the main hypothesis: Training SC improves the psychological well-being of infertile women 

To examine the main hypothesis of the research, covariance analysis was used. Before the test, the assumptions were investigated. The results indicated a normal distribution of data, the homogeneity of the variance, the reliability of the covariate variable, performing the pre-test before starting the study and the intervention, and the homogeneity of the slope regression.

According to the (table IV), there was a significant difference between the estimated mean scores for improving the psychological well-being of the participants in the experiment and control groups (P = 0.01), and the difference indicates that 72.7% of the covariance of the post-test scores is due to SC intervention. Therefore, the intervention of SC training affects the improvement of psychological well-being among infertile women. Also, the pre-test variable is significant with the effect of 94.2% (P = 0.01).

**Table 2:** Descriptive statistics of psychological well-being variables in infertile women in the control and experiment groups


**Group**	**Minimum**	**Maximum**	**Mean ± SD**
Control	Pre-test	276	383	317.38 ± 42.682
	Post-test	280	387	320.88 ± 40.186
Experiment	Pre-test	278	387	316.94 ± 42.496
	Post-test	299	404	349.88 ± 36.648

**Table 3:** Comparison of the psychological well-being of infertile women in the control and experiment groups


**Group**	**Pre-test**	**Post-test**	P-value*
Control	317.38	320.88	0.15
Experiment	316.94	349.88	0.01
P-value**	0.98	0.04	
*Paired sample t-test; **independent sample t-test			

**Table 4:** Analysis of covariance (ANCOVA) to study the effect of educational intervention on the psychological well-being of infertile women in the post-test phase


**Source**	**Type III sum of squares**	**df**	**Mean square**	**F**	P-value*	**Partial eta squared**
Psychological well-being pretest	41779.933	1	41779.933	467.884	0.01	0.942
Group	6906.859	1	6906.859	77.348	0.01	0.727
Error	2589.567	29	89.295		
*ANCOVA						

## 4. Discussion

The main hypothesis of this study was to investigate the effectiveness of SC training on the psychological well-being of infertile women, which the results of this study support this hypothesis. In other words, SC entails the following three main components: (a) self-kindness, (b) common humanity, and (c) mindfulness that have an influence on the structure of psychological well-being, which includes dimensions such as self-acceptance, having positive relationships with others, autonomy, environmental dominance, personal development, and having a goal in life. The findings of this study have been consistent with several studies discussed as follows:

Neff and Pommier revealed that SC can improve interpersonal performance and is associated with characteristics such as empathy and altruism (9). Also, in another studies' findings, which examined the effect of the mindfulness-based program on infertile women, showed that boosting mindfulness and skill of acceptance, as well as cognitive seperation from negative thoughts and feelings, appear to help women to experience negative inner states in new ways, decreasing their entanglement with them and thus their psychological distress (12). Similarly, the results of another study, which examined mindfulness, SC, and psychological well-being in patients with chronic depression, showed that patients who had been trained mindfulness and SC for six months had less replication with negative thoughts and judged themselves less. The results of mentioned study showed the importance of positive psychology in comparison with damage-based psychology (13). An investigation about the relationship between mindfulness, SC and depression symptoms in infertile women showed that there is a positive and significant relationship between the dimensions of mindfulness and SC, including not judging experiences, knowingly acting, observing, and describing the symptoms of pre-natal depression (14).The findings of the present study and literature review indicate that SC training has an impact on emotion regulation (15), and it has a significant relationship with emotional intelligence. From the medical point of view, with increasing SC in people and reducing the negative emotions, the secretion of the oxytocin hormone in the brain increases, which has a direct impact on the reduction of amygdala risk. Since the purpose of compassion-focused therapy is the gradual stimulation of the emotional-focused system cognitive training, mindfulness-based program, and relaxation are used to promote mental health of individuals.

## 5. Conclusion

In brief, the results of this study show that compassion based training has a significant role in infertile females' mental health. As this study's finding show the powerful effect of self-compassion training on well-being of infertile female.

##  Conflict of Interest 

The authors declare that they have no competing interest.
